# Author’s Response to Peer Reviews of “Comparison Between Male and Female Survivors of Sexual Abuse and Assault in Relation to Age at Admission to Therapy, Age of Onset, and Age at Last Sexual Assault: Retrospective Observational Study”

**DOI:** 10.2196/34622

**Published:** 2021-11-26

**Authors:** Ali M AL-Asadi

**Affiliations:** 1 Department of Arts and Education Grande Prairie Regional College Grande Prairie, AB Canada; 2 Department of Psychology University of South Africa Johannesburg South Africa

**Keywords:** sexual abuse, sexual assault, age of onset, sex, gender, age, therapy, abuse, assault, mental health, victim, childhood, children, gender disparity, violence

*This is the author’s response to peer-review reports for “Comparison Between Male and Female Survivors of Sexual Abuse and Assault in Relation to Age at Admission to Therapy, Age of Onset, and Age at Last Sexual Assault: Retrospective Observational Study.”*

## Round 1 Review

### Major Comments

1. Corrected [1]. The sections have been switched around [2] and the bullet format was changed to paragraphs.

2. More texts and references to previous studies were added where applicable.

3. A statistical metric (Pearson *r*) was reported where a linear relationship was mentioned.

### Minor Comments

1. Both are corrected.

2. I shall consider bootstrapping methods in future submissions where applicable.

Finally, a comment to the editor: I used Mendeley's AMA 11th edition style.

Thank you so much for the reviewer's work and your diligence.
